# Prevalence and interference of neuropathic pain in the quality of life in patients with knee osteoarthritis

**DOI:** 10.1016/j.clinsp.2023.100287

**Published:** 2023-09-29

**Authors:** Camilo Partezani Helito, Fernando Sant'Anna Moreira, Matheus Augusto Maciel Santiago, Lucas de Faria Barros Medeiros, Pedro Nogueira Giglio, Andre Giardino Moreira da Silva, Riccardo Gomes Gobbi, José Ricardo Pécora

**Affiliations:** a Grupo de Joelho, Instituto de Ortopedia e Traumatologia, Hospital das Clínicas, Faculdade de Medicina, Universidade de São Paulo (HCFMUSP), São Paulo, SP, Brazil; b Hospital Sírio Libanês, São Paulo, SP, Brazil

**Keywords:** Neuropathic pain, Knee osteoarthritis, Quality of life, Gonarthrosis, Osteoarthritis

## Abstract

**Objective:**

With the aging of the population, more patients have complained of pain due to knee Osteoarthritis (OA), and the number of arthroplasties has also increased. The objective of this study is to evaluate the prevalence of the neuropathic pain component in candidates for Total Knee Replacement and the effects of this component on their quality of life.

**Methods:**

In this cross-sectional study, patients with OA candidates for knee arthroplasty in the present institution were evaluated using the pain detection questionnaire and the Visual Analog Pain (VAS) scale to measure the pain index and the presence of associated neuropathic pain. In addition, evaluation of the quality of life and functionality using the EQ5D and SF12 questionnaires and their relationship with cases of neuropathic pain were performed.

**Results:**

One hundred twenty-six patients were evaluated, and 71.4 % were female. The age ranged from 46 to 85 years, and about 70 % of the patients had some associated clinical comorbidity. Neuropathic pain was present in 28.6 % of the patients evaluated. Patients with neuropathic pain presented worse results in the VAS evaluation, in the care, pain, and anxiety domains of the EQ5D, and in the physical and mental scores of the SF12.

**Conclusion:**

Neuropathic pain was present in 28.6 % of the patients with knee OA who are candidates for arthroplasty. Patients with associated neuropathic pain present a higher level of pain and worse quality of life scores. Recognizing this type of pathology is extremely important in fully monitoring gonarthrosis.

## Highlights

• Total Knee Arthrosplasty (TKA) candidates often have associated neuropathic pain.
• Patients with gonarthrosis and neuropathic pain have worse functional scores.
• TKA candidates should be assessed for the presence of associated neuropathic pain.

## Introduction

With the aging of the population worldwide, Osteoarthritis (OA) is already the most prevalent musculoskeletal disease and the main cause of chronic motor dysfunction in developed countries. Epidemiological studies indicate an increasing incidence, which today reaches approximately 15 % worldwide, making the condition a disease that greatly impacts society, leading to early retirement and high costs for the health system [1,2].

Osteoarthritis is one of the most frequent causes of chronic pain [1,3]. The pathophysiology of pain mainly involves nociceptive and neuropathic mechanisms. The pathological alterations of OA involve degeneration of the hyaline articular cartilage, bone involvement with remodeling, and synovial hyperplasia leading to chronic inflammation that can generate alterations of the peripheral nervous system, affecting the nociceptive afferent signals of the joint as a whole [1], [2], [3], [4]. Neuropathic pain has been increasingly studied and accepted within the context of OA. It can happen due to an injury or dysfunction of the central or peripheral nervous system. Peripherally, the subchondral bone, rich in sensory nerve fibers, is affected in the more advanced stages of OA, developing a neuropathic pain component [5,6]. In the central nervous system, neuromodulation and plasticity phenomena can generate neuropathic pain, evidenced by the efficacy of duloxetine, a centrally acting drug, in patients with knee OA [7,8]. Cases of chronic pain can also lead to a hypersensitization mechanism, generating a persistent high reactivity that potentiates the painful status [9].

Recent studies have identified a prevalence of approximately 15 %–25 % of patients with neuropathic pain associated with OA [10,11]. Often patients can maintain postoperative pain and have poor results after surgery, due to an incorrect diagnosis and treatment of the neuropathic pain, even in cases in which the surgical technique was adequate, which may persist and even worsen after surgery. DeFrance and Scuderi [12] showed that factors such as pain at rest, low back pain, and pain in multiple joints could be related to persistent knee pain after knee arthroplasty. These factors may also be associated with neuropathic pain. Studies with the empirical use of pregabalin, a medication classically used in treating chronic neuropathic pain components, have shown the efficacy of this medication in perioperative knee arthroplasty [13].

In clinical practice, the assessment of neuropathic pain components in patients with OA candidates for Total Knee Arthroplasty (TKA) is not performed routinely. Thus, the objective of this study is to evaluate the prevalence of the neuropathic pain component in candidates for TKA and the effects of this component on their quality of life. In addition, to compare the demographic characteristics of patients with and without neuropathic pain components. The authors hypothesize that the neuropathic pain will be frequent and will cause functional impairment in patients.

## Methods

### Participants

This cross-sectional study selected and interviewed patients candidates for TKA who are waiting for the procedure in the present institution. The study was approved by the Institution's Ethics Committee, with reference number 41909021.7.0000.0068, and the informed consent form was obtained from the participants.

The inclusion criterion was the indication for TKA without age limits. The exclusion criteria were previous surgical procedures in the knees, patients with cognitive impairment, therefore, unable to answer the questionnaires, and known pathology of the central or peripheral nervous system that could interfere with the neuropathic pain evaluation.

All patients included were already being followed up by the service under conservative treatment, including physical rehabilitation and analgesic drugs. The interviews were conducted by two physicians, one first-year resident and another physician specializing in Orthopedics and Traumatology, specializing in knee surgery.

The study was conducted by telephone or personal interviews during outpatient visits. The following data were collected: epidemiological data such as age, gender, and associated comorbidities; pain time (considered as the first time that the patient reported knee pain in a lasting manner and with impairment in quality of life); date of indication for the surgical procedure (waiting time for TKA); Visual Analog Pain (VAS) scale; pain detect questionnaire, to assess the presence of neuropathic pain; evaluation by EuroQol-5 Dimensions Questionnaire (EQ-5D) and by the 12-Item Short-Form Health Survey revised form (SF-12v2).

From the questionnaire's application, 150 patients were initially evaluated for neuropathic pain through the pain detection questionnaire*.* The questionnaire scores range from 0 to 38 points. Scores between 0 and 12 are considered nociceptive pain, between 13 and 18 are considered unclear or possible for neuropathic pain component, and scores ≥ 19 represent a high probability of neuropathic pain component. Patients with unclear scores were excluded from the analysis to avoid possible bias as due to the complexity of the accurate diagnosis of the neuropathic component, it would be illogical to include patients with an unclear diagnosis in any of the studied groups.

### Data analysis

Data were expressed as mean and Standard Deviation (± SD) and median and interquartile range for continuous variables and as absolute numbers and percentages for categorical data.

The comparison between groups was performed using Pearson's Chi-Square test or Fisher's exact test for categorical variables. The differences between groups for continuous variables were calculated using the *t*-test or Mann-Whitney *U* test.

The variables that could be associated with the physical score and mental score status (dependent variables) were evaluated using the Multiple Linear Regression (MLR) model and performed by the statistical software SPSS; p-values < 0.05 were considered significant.

No sample size calculation was initially performed, as all patients that were available for inclusion were included. Therefore, the authors used a convenience sample at the beginning, but the post-hoc power analysis showed a study power of 99 % for the VAS evaluation between groups with a high and low probability of neuropathic pain.

## Results

Neuropathic pain based on the pain detect questionnaire was present in 28.6 % of the patients evaluated (36 cases) (Table 1); pain with a low probability of neuropathic pain component was seen in 90 patients and another 34 were considered as unclear and, therefore, excluded from the final analysis. From the final population studied (*n* = 126), 71.4 % were female and 28.6 % male, the age of the participants ranged from 46 to 85 years, and about 70 % of the patients had some clinical comorbidity. An incidence of 69 % Systemic Arterial Hypertension (SAH), 18 % Diabetes Mellitus (DM), and approximately 8 % Rheumatoid Arthritis (RA) were observed. Other comorbidities such as dyslipidemia, thyroidopathy, mood disorder, or asthma were present in 26.9 % of patients.

**Table 1:** Epidemiological characteristics of the study population (*n* = 126).

Variables		*n* = 126	%
Sex	Female	90	71.4
Age (years)	Mean	67.5	
Comorbidities	Yes	89	70.6
DM	Yes	23	18.3
SAH	Yes	87	69
RA	Yes	10	7.9
Other comorbidities	Yes	34	26.9
Neuropathic pain	Yes	36	28.6

DM, Diabetes Mellitus; SAH, Systemic Arterial Hypertension; RA, Rheumatoid Arthritis.

There were no differences regarding epidemiological data and clinical comorbidities when comparing the group with and without associated neuropathic pain (Table 2). However, patients with neuropathic pain presented a higher VAS (8.06 vs. 6.41; *p* < 0.001), with a difference greater than 1.5 points compared to patients without a neuropathic pain component. In the EQ5D score, of the five existing domains surveyed, patients with neuropathic pain presented worse results in daily care, pain, and anxiety. Although the other two domains (mobility and activities of daily living) did not show statistical significance, the neuropathic pain group also presented worse results in absolute numbers. Reinforcing the results obtained in the other questionnaires, the results of the SF-12v2 after calculations of the physical and mental scores also revealed inferiority in the neuropathic pain group (Table 3).

**Table 2:** Epidemiological characteristics according to the neuropathic pain component (*n* = 126).

Variables		Pain with low probability of neuropathic pain component	Pain with a probable neuropathic pain component	*p*
		*n* = 90 (%)	*n* = 36 (%)	
Sex	Female	60 (66.7)	30 (83.3%)	0.061
Age (years)	Mean (SD)	68.1 (8.1)	66 (9.4)	
Comorbidities	Yes	63 (70)	26 (72.2)	0.805
DM	Yes	17 (18.9)	6 (16.7)	0.771
SAH	Yes	62 (68.9)	25 (69.4)	0.951
Mood disorders	Yes	6 (6.7)	2 (5.6)	1
Obesity	Yes	1 (1.1)	0 (0)	1

DM, Diabetes Mellitus; SAH, Systemic Arterial Hhypertension.

**Table 3:** Clinical outcomes according to the neuropathic pain component (*n* = 126).

Variables		Pain with low probability of neuropathic pain component	Pain with a probable neuropathic pain component	*p*
		*n* = 90 (%)	*n* = 36 (%)	
Pain time (months)	Mean	127	131.1	0.793
VAS	Mean (SD)	6.41 (2.42)	8.06 (1.72)	<0.001
PAIN DT Score	Mean (SD)	6.68 (3.6)	23 (3.26)	<0.001
EQ Mobility	Mean (SD)	1.98	2.03	0.183
EQ Care	Mean (SD)	1.57 (0.6)	1.92 (0.6)	0.004
EQ Activities	Mean (SD)	2.09	2.17	0.387
EQ Pain	Mean (SD)	2.37 (0.5)	2.72 (0.45)	<0.001
EQ Anxiety	Mean (SD)	1.89 (0.69)	2.17 (0.69)	0.042
EQ Score	Mean (SD)	21578.89 (2808.38)	22440.5 (1791.46)	0.091
Physical Score	Mean (SD)	28.94 (8.42)	25.32 (6.67)	0,023
Mental Score	Mean (SD)	46.31 (11.87)	41 (11.64)	0.024

SD, Standard Deviation.

The results of multivariate analysis and logistic regression are summarized in Tables 4 and 5. The factors associated with neuropathic pain in logistic regression were VAS and physical and mental scores. The variables related to a worse physical score, identified by multiple linear regression, were pain time, VAS, and EQ score.

**Table 4:** Multivariate analysis of factors associated with neuropathic pain component (*n* = 126).

Neuropathic pain component			
Variables	OR	95% CI	*p*
VAS	1.37	1.08–1.74	0.009
Physical score	0.91	0.84–0.99	0.026
Mental score	0.94	0.90–0.98	0.006
Comorbidity	1.02	0.39–2.63	0.969
EQ score	1.00	1.00–1.00	0.237

OR, Odds Ratio; CI, Confidence Interval.

**Table 5:** Results of multiple linear regression analysis applied to the 126 patients. Physical score used as the dependent variable.

Physical score			
Independent variables	β	*t*	*p*
Comorbidity	0.085	1.053	0.295
Pain time	−0.182	−2.257	0.026
VAS	−0,358	−4.424	0.000
EQ score	−0.210	−2.599	0.011

Coefficient of determination (R²) = 0.216; adjusted R² = 0.191; multiple correlation coefficients = 0.465; residual standard deviatio *n* = 7.292; F = 8.356 (*p* < 0.001).

## Discussion

The main finding in this study was the associated neuropathic pain component of 28.6 % in cases of knee OA waiting for TKA. In addition, cases with associated neuropathic pain presented greater pain intensity and worse quality of life scales, confirming the initial hypothesis.

The prevalence of neuropathic pain in cases of OA found in this study is consistent with the previous literature. In a recent systematic review with meta-analysis, Zolio et al. found a prevalence of neuropathic pain by the pain detection questionnaire at 20 % for knee OA and 9 % for hip OA [14]. In the study conducted by Ohtori et al., the neuropathic pain component was only 5.4 %, but the patients with this associated condition presented worse VAS and WOMAC scales [10]. In the present study, the neuropathic pain component was almost 30 %, and these cases also presented higher pain scores according to the VAS assessment.

Regarding the quality of life by EQ5D, among the five variables surveyed, the results showed a negative impact on quality of life in cases of associated neuropathic pain components, especially those related to daily care, pain, and anxiety. Ibor et al. conducted a study including more than 5000 patients who sought orthopedic services for pain assessment [15]. Patients with associated neuropathic pain presented worse health status, greater clinical complexity, higher comorbidities, and poorer quality of life. The evaluation of populations with neuropathic pain systematically shows a worse quality of life in the most diverse orthopedic pathologies, including OA. In the study by Ibor et al., neuropathic pain in cases of OA (not only knee OA) was 29 %, very similar to the present study. As a comparison, in the lower back or cervical pain cases, the neuropathic pain component reaches 54 % and 30 %, respectively.

The association of neuropathic pain with limited quality of life is cited by the National Institute for Health and Care Excellence [16] and was also observed in the present study. In the SF12v2 questionnaire, a tendency to worsen the patient's quality of life and perception of health in the neuropathic group was noticed. Using a validated calculator for data obtained in SF12v2, the physical and mental scores for each group were found. From the statistical analysis of parametric data, worse physical (*t* = 0.023) and mental (*t* = 0.026) scores were found with statistical significance, reinforcing the limitation of quality of life due to the neuropathic pain component. This questionnaire also evaluates factors related to the patient's social life, which is also impaired. These factors may also be related to a non-diagnosis of neuropathic pain in the orthopedic evaluation, leading to inadequate treatment. As there is no well-established diagnostic test and the diagnosis is based on a set of history and physical examination factors, the neuropathic component is often not diagnosed and therefore is also not treated [17].

The proportion of patients with residual pain after TKA can vary from 8 % to 27 % [18], with the neuropathic pain component possibly responsible for this postoperative outcome. Recent studies show that, although TKA substantially decreases the prevalence of neuropathic pain in OA patients, the presence of preoperative neuropathic pain may indicate unfavorable postoperative evolution [19]. DeFrance and Scuderi [12] did a recent systematic review of the post-TKA dissatisfaction rate. The results showed that 7 % to 16 % of patients are dissatisfied after arthroplasty, and the sociodemographic factors that lead to this outcome are diverse. The study did not evaluate patients specifically according to the presence or absence of neuropathic pain; however, factors often associated with neuropathic pain, such as pain at rest, associated low back pain, intense and disproportionate pain, pain in multiple joints, and pain in joints other than the knee, were common factors. This patient dissatisfaction and the incomplete pain improvement in the postoperative may result from hyperexcitation or hyper-responsiveness to pain stimulus and changes in pain modulation, resulting from intense and poorly controlled pain over time during the period in which the patient treated the OA non-operatively. Identifying these patients early and treating the neuropathic pain component may be necessary to minimize unfavorable postoperative outcomes. Patients with neuropathic pain may have a slower recovery regarding pain control and gain of range of motion [20], [21], [22].

Considering the findings of this study, the authors believe it is very important to systematically evaluate the neuropathic pain in patients candidates for TKA to minimize the possibilities of unfavorable results after surgery. Protocols to manage risk factors such as diabetes and inflammatory diseases, known to increase the risks of complications and unfavorable outcomes after TKA, already exist and are well established [23], [24], [25]. Therefore, neuropathic pain should also be part of the preoperative evaluation of TKA candidates.

The study's limitations include the study design (cross-sectional), which is subject to prevalence biases, and, as the data collected and outcomes were evaluated at a single moment, it is difficult to establish an exact temporal relationship between them. However, the relationship between the neuropathic pain component and OA presents plausibility and consistency, which increases the chance of causality between them. In addition, the present study did not have the primary objective of establishing the relationship between the neuropathic pain component and OA but rather estimated their coexistence and how this interferes with OA therapeutic decisions. Also, the study relies on self-report measures for data collection, which may introduce recall bias and subjective interpretation by the participants finally, the study was conducted in a single institution, which may limit the generalizability of the findings to other healthcare settings or patient populations with different demographics or healthcare access. The fact that patients 34 patients were excluded from the final analysis due to scoring points that fall in the unclear area of the pain detection questionnaire may also be considered a limitation, however, due to the complexity of the diagnosis of this pathology, it would be inaccurate to move this population to any of the other two groups of interest. Further studies evaluating only this population could be performed in the future to verify how it behaves.

## Conclusion

Neuropathic pain was present in 28.6 % of the patients with knee OA who are candidates for arthroplasty. Patients with associated neuropathic pain present a higher level of pain and worse quality of life scores. Recognizing this type of pathology is extremely important in fully monitoring gonarthrosis.

## Authors' contributions

Camilo Partezani Helito: Conceptualization, Writing – original draft. Fernando Sant'Anna Moreira: Data curation, Writing – original draft. Matheus Augusto Maciel Santiago: Data curation, Resources. Lucas de Faria Barros Medeiros: Data curation, Resources, Writing – review & editing. Pedro Nogueira Giglio: Formal analysis, Methodology. Andre Giardino Moreira da Silva: Writing – review & editing. Riccardo Gomes Gobbi: Supervision, Project administration, Methodology. José Ricardo Pécora: Supervision, Project administration, Writing – review & editing. All authors read and approved the final manuscript.

## Ethics approval

This study was performed in line with the principles of the Declaration of Helsinki. Approval was granted by the Ethics Committee of the University of São Paulo (CAAE 41909021.7.0000.0068; 15/02/2021).

## Declaration of Competing Interest

The authors declare no conflicts of interest.
